# ESCRT machinery: role of membrane repair mechanisms in escaping cell death

**DOI:** 10.1038/s41392-022-01108-6

**Published:** 2022-07-16

**Authors:** Lisa Gregor, Sophia Stock, Sebastian Kobold

**Affiliations:** 1 Division of Clinical Pharmacology, Department of Medicine IV, University Hospital, Ludwig Maximilian University (LMU) of Munich, Lindwurmstrasse 2a, 80337 Munich, Germany; 2 Department of Medicine III, University Hospital, Ludwig Maximilian University (LMU) of Munich, Marchioninistrasse 15, 81377 Munich, Germany; 3 German Cancer Consortium (DKTK), Partner Site Munich, Pettenkoferstrasse 8a, 80336 Munich, Germany; 4 Einheit für Klinische Pharmakologie (EKLiP), Helmholtz Zentrum München, German Research Center for Environmental Health (HMGU), Ingolstädter Landstrasse 1, 85764 Neuherberg, Germany

**Keywords:** Tumour immunology, Adaptive immunity

A recent research article published in *Science* by Ritter et al. reported that endosomal sorting complexes required for transport (ESCRT) proteins mediate repair of lesions in the cell membrane caused by the pore-forming toxin perforin at sites of cytotoxic T cell engagement.^1^ Subsequent entry of granzymes and initiation of apoptosis of cancer cells is thus limited, leading to reduced sensitivity against T cell‑mediated cytotoxicity.^1^ Along these lines, this could constitute a new critical resistance mechanism for T cell-based treatment approaches.

Cellular immunotherapy has gained more relevance in recent years regarding the treatment of various cancers. This therapeutic approach takes advantage of tumor cell killing mediated by cytotoxic lymphocytes including cytotoxic T cells and natural killer (NK) cells. During an immune response, cytotoxic T cells and NK cells can induce apoptosis of target cells through death receptor-mediated cytotoxicity, involving the death receptor ligands, tumor necrosis factor-related apoptosis-inducing ligand and Fas ligand, or by granule-mediated cytotoxicity, involving perforin and granzymes.^2^ During granule-mediated cytotoxicity, cell lysis is induced after tumor antigen recognition by secretion of cytotoxic pore-forming perforin. Subsequent osmotic flux through the membrane pores leads to cell swelling and necrotic cell death.^2^ Perforin enables the uptake of cell death inducing granzymes through diffusion or endocytosis.^2^ Interestingly, recent studies have suggested that cellular killing mediated by such enzymes is an additive multi-step process, whereby multiple rounds of granule secretion are necessary to induce apoptosis of the target cell.^3^ This however can be compensated by target cells themselves by repairing such pores in the cellular membrane.^1^

The ESCRT machinery plays a key role during extracellular budding events necessary for repair of perforin-induced pores.^4,5^ Previous studies have shown that membrane pore formation leads to an increase in calcium influx, as well as a drop in membrane tension, which in return triggers the recruitment of the ESCRT machinery to the site of membrane wounding.^4,5^ However the contribution of the ESCRT machinery to tumorigenesis is still controversial, as multiple experimental approaches in different models revealed partially divergent conclusions.^6^ Besides the defective transmembrane protein homeostasis due to membrane pore formation and the consequent effect on cell signaling, also cytokinetic defects lead to a rather tumorigenic potential.^6^ It was shown that ESCRT-mutated cells fail to downregulate and degrade cell-surface signaling receptors, leading to a prolonged receptor signaling and hyperproliferation in tissues.^7^ Contrarily, increased apoptosis and cell-cycle defects might counteract this hyperproliferation.^7^ Furthermore it has been shown that the ESCRT machinery is involved in exosome generation and autophagy, whereby ESCRT mutated cells show defects in such processes.^6^ Ultimately, better understanding of these repair mechanisms in the context of cancer development will allow to specifically inhibit these processes and by such prevent tumor cell escape.

Here, Ritter et al. investigated the recruitment of the ESCRT machinery to sites of cytotoxic T cell engagement after perforin secretion.^1^ Live-cell microscopy of cytotoxic T cells derived from OT-1 mice, recognizing the ovalbumin peptide SIINFEKL on target cells, were used to model this situation. For better visualization, parts of the ESCRT machinery expressed enhanced green fluorescent protein (EGFP) tags. Interaction of cytotoxic T cells with target cells was analyzed in presence of the cell impermeable fluorogenic dye propidium iodide (PI), which, after diffusion through perforin pores, can bind and illuminate nucleic acids in the nucleus and cytosol. The researchers demonstrated that the EGFP-tagged ESCRT proteins were recruited to the site of T cell-target cell contact. To further assess the immunological synapse immediately after secretion of lytic granules, highly sophisticated microscopy techniques were applied. It could be confirmed that the ESCRT machinery is located within the synapse at the site of cytotoxic T cell killing. Therefore, the accumulation of ESCRT proteins near membrane pores is linked to perforin secretion.

**Fig. 1:**
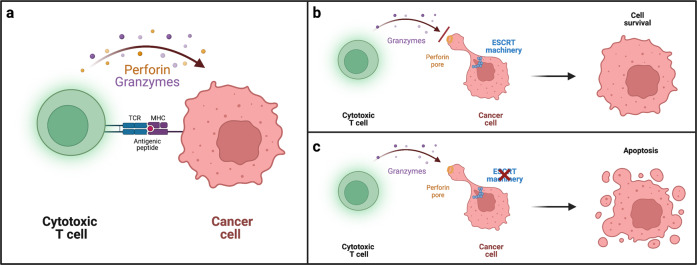
**a** Schematic illustration of secretion of the pore-forming toxin perforin and the following delivery of apoptosis-inducing serine proteases (granzymes). **b** Membrane repair of perforin pores by the ESCRT machinery limits granzyme entry into the cytosol, allowing target cells to better resist the cytolytic attack. **c** Inhibition of the ESCRT machinery in cancer cells increases their susceptibility to cytotoxic T cell-mediated killing

In a next step, the authors could show that the cytotoxic capacity of OT-1 T cells was enhanced in ESCRT-inhibited target cells, which were more sensitive to perforin- and granzyme-mediated killing. Delays in pore repair also increased uptake of granzyme B. Thus, ESCRT-inhibited target cells were more sensitive to the cytotoxic effect of perforin and granzyme B. A schematic illustration of the role of membrane repair by the ESCRT machinery for resistance of tumor cells to cytotoxic T cell killing is summarized in Fig. 1.

With this work, the authors have provided detailed spatial and temporal information on membrane repair via the ESCRT machinery by using highly advanced imaging technologies.^1^ Further application of a human assisted machine learning-computer vision platform allowed for the visualization of T cell-mediated killing, enabling subcellular resolution of the immunological synapse. Through the biological impairment of the ESCRT function, the authors could demonstrate an increased T cell killing capacity of target cells.^1^ This showcases the importance of the ESCRT machinery for cancer cells to escape T cell-mediated apoptosis, which constitutes a hallmark of cancer. It is important to point out however, that the demonstrated mechanism is restricted to murine cell models and artificial model antigen systems. Further investigations are required to identify if the ESCRT machinery holds a similar pivotal role in human cancer and immune cells, a fundamental question for therapeutic utilization.

This work illustrated increased target cell killing when inhibiting the ESCRT machinery.^1^ However, a CRISPR knockout or overexpression of a dominant-negative kinase allele impairing the ESCRT function were used for ESCRT inhibition, which are not reasonably feasible for clinical application. To further enhance the translational potential of these findings, the development of specific small-molecule compounds to target membrane-repair pathways would be beneficial. Regarding the fact that membrane-repair mechanisms also exist in non-malignant cells, potential short and long-term side effects of ESCRT-targeted therapy in cancer treatment would need further evaluation. As the multi-subunit ESCRT machinery is composed of numerous proteins, it is unclear whether individual components of the machinery affect tumor cell survival in different manners in comparison to healthy cells.^6^ Such discriminatory capacity would be pivotal for the application of ESCRT-inhibition.

The field of immunotherapy is largely focusing on optimization of anti-cancer toxicity mediated by immune cells, such as cytotoxic T cells and NK cells. Despite major advances, immunotherapy does not benefit most patients in the long run, whereby underlying causes are frequently unclear and contrast expectations from preclinical work. The concept of using T cell-mediated cytotoxicity for cancer treatment is indeed supported by the clinical application of immune checkpoint inhibitors, bispecific antibodies, or genetically modified T cells. Along these lines, any resistance mechanism preventing T cell action, will ultimately hamper therapeutic function. Thus, targeting the ESCRT machinery to render cancer cells more susceptible to perforin-mediated cell killing might facilitate a new targeting approach to extend the damage caused by individual cytotoxic T cells. To our knowledge the combination of T cell-based immunotherapy and interference with the ESCRT machinery in such manner is not yet thoroughly investigated.

Besides identification of more potential links between cancer and the ESCRT machinery, the complete role of ESCRT inhibition for cancer is still not fully understood. A critical need to understand resistance mechanisms to key immunological approaches remains. The study presented by Ritter et al. provides valuable insights into the membrane repair mechanisms at the immunological synapse, which could be used to enhance efficacy of T cell-based immunotherapy, warranting further investigations and potential clinical translation of the concept.
